# Hydrogen Embrittlement of Boron-Modified Supermartensitic Stainless Steel

**DOI:** 10.1021/acsomega.5c02404

**Published:** 2025-08-11

**Authors:** Arthur de Bribean Guerra, Daniel Brito Bertoldi, Guilherme Zepon, Paulo Sérgio Carvalho Pereira da Silva, Tomaz Toshimi Ishikawa, Guilherme Yuuki Koga, Claudemiro Bolfarini

**Affiliations:** † Department of Materials Science and Engineering, 67828 Federal University of São Carlos, Rod. Washington Luís, São Carlos, SP CEP 13565-905, Brazil; ‡ Graduate Program in Materials Science and Engineering, 67828 Federal University of São Carlos, Rod. Washington Luís, São Carlos, SP CEP 13565-905, Brazil; § Center for Characterization and Development of Materials (CCDM), 67828 Federal University of São Carlos, Rod. Washington Luís, São Carlos, SP CEP 13565-905, Brazil

## Abstract

Supermartensitic stainless steels (SMSS) reinforced with a percolated boride network offer exceptional corrosion and wear resistance, making them well-suited for oil and gas applications. However, hydrogen embrittlement (HE) poses significant challenges in offshore environments. This study examines HE in SMSS with boron additions ranging from 0.3 to 0.7 wt %. Samples were subjected to hydrogen charging at −1.3 VSCE, 43 mA/cm^2^ for 168 h in a 3.5 wt % NaCl solution, followed by slow strain rate tensile testing. While boron additions improved tensile strength, they severely reduced ductility. After hydrogen charging, mechanical degradation intensified, with SMSS-0.7%B experiencing >55% reduction in tensile strength and elongation. All boron-rich alloys displayed brittle intergranular fractures caused by M_2_B borides at the grain boundaries. HE impacted both the martensitic matrices and borides, with fractures initiating in boride-rich regions and propagating via secondary cracks. These results underscore a synergistic embrittlement mechanism and emphasize the importance of optimizing microstructures to enhance hydrogen resistance in boron-reinforced SMSS.

## Graphical abstract

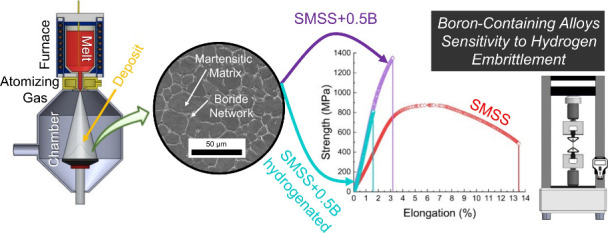

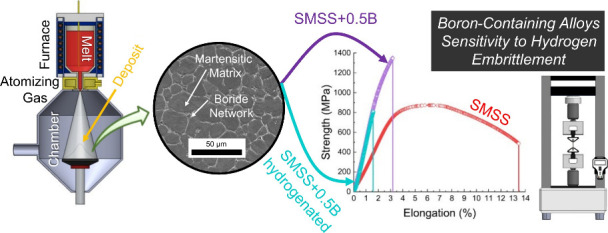

## 1 Introduction

Cracks in offshore structures and pipelines can lead to catastrophic failures, often associated with hydrogen-induced cracking (HIC) in aggressive environments.^1^ The increasing demand for deep-sea oil extraction, particularly from presalt reservoirs at depths exceeding 2 km, exposes critical equipment to extreme operational conditions, including high pressures, severe corrosion, intense wear, and hydrogen embrittlement (HE).^2^ HE is primarily driven by hydrogen sulfide (H_2_S) exposure, cathodic protection methods, and welding processes, all of which facilitate hydrogen ingress into metallic structures.^3–5^

Hydrogen’s small atomic size and high diffusivity enable it to penetrate metallic materials, where it accumulates at defects, grain boundaries, and other microstructural inhomogeneities. This accumulation weakens interatomic bonds, promotes crack initiation, and accelerates crack propagation.^6,7^ The detrimental effects of HE include reduced ductility, altered fracture morphology, and significantly decreased toughness, all of which compromise the structural integrity of offshore components.^8^ Supermartensitic stainless steels (SMSS) have been widely adopted in offshore applications, such as risers, pipelines, and drill columns, due to their exceptional strength and corrosion resistance.^9–12^ However, their high susceptibility to HE, particularly under dynamic loading and aggressive environments, has led to premature failures, raising concerns about their long-term reliability.^13,14^

To address wear-related degradation, boron-added spray-formed SMSS has been developed. This innovative material leverages the high hardness and wear resistance imparted by boron while retaining the inherent corrosion resistance of stainless steel matrices.^15–17^ Despite these advancements, the mechanical behavior of these alloys under hydrogen exposure remains poorly understood. A deeper understanding of the interactions between hydrogen and the modified microstructures of SMSS is critical for ensuring the reliability and safety of offshore applications. Various mitigation strategies such as surface coatings, alloy modifications, and hydrogen trapping mechanisms have been explored to enhance resistance to HE.^18^

Recent advances in electrochemical hydrogen charging, *in situ* fracture analysis, and multiscale modeling techniques have provided new insights into the mechanisms of hydrogen-induced material degradation.^19^ While computational models offer valuable predictive capabilities, experimental validation remains essential for accurately assessing hydrogen interactions with microstructural features.^20,21^ Furthermore, the development of advanced testing methodologies, such as high-pressure hydrogen environments and real-time monitoring systems, is crucial for designing materials with improved resistance to HE.

This study aims to investigate the impact of HE on the mechanical properties and fracture behavior of boron-modified SMSS, providing critical insights into their performance under extreme offshore conditions. A comprehensive approach combining microstructural characterization, fracture analysis, and electrochemical testing was employed to evaluate the suitability of these materials for offshore applications. The findings are expected to contribute to the development of next-generation stainless steels with enhanced durability and reliability in hydrogen-rich environments, addressing a critical need for safer and more efficient offshore operations.

## 2 Materials and Methods

Three supermartensitic stainless steel (SMSS) alloys were produced with target boron additions of 0.3, 0.5, and 0.7 wt %. The contents of Mo, Cr, and Ni were kept similar to the SMSS. Raw materials included SMSS and iron–boron alloy (16 wt % B). To account for the boron addition, iron–molybdenum alloy (62 wt % Mo), pure chromium, and nickel were added. Approximately 4 kg of each composition was melted in an induction furnace, atomized with nitrogen gas (0.5 MPa), and deposited onto a rotating carbon steel substrate (250 mm diameter) at 1650 °C, with a flight distance of 460 mm and a substrate rotation of 45 rpm.

The alloy compositions were checked by inductively coupled plasma atomic emission spectrometry (ICP-AES), while C and S were analyzed by direct combustion in a LECO CS-844. O, N, and H contents were measured via an inert gas fusion thermal conductivity method using a LECO ONH-836 instrument. Microstructures were characterized using optical microscopy and scanning electron microscopy (SEM) by using a Philips XL30 FEG instrument equipped with energy-dispersive spectroscopy (EDS) and X-ray diffraction (XRD). Samples were microetched with aqua regia for microstructural analysis.

Tensile specimens were machined (ASTM E8) and exposed to hydrogenation in a 3.5 wt % NaCl electrolyte, being tested using an Instron 5585H apparatus. A platinum electrode served as the anode, and a current density of 43 mA/cm^2^ was applied for up to 168 h at 25 °C. These hydrogen charging conditions were selected based on prior literature for stainless steels.^22,23^ Samples were isolated with inert tape, leaving only the 16 mm gauge length exposed. Tensile tests were conducted on hydrogenated and nonhydrogenated samples at a slow strain rate of 1.6 × 10^–4^ s^–1^, following ASTM E8 ^24^ and ASTM G129.^25^ Fracture surfaces, as well as polished/etched longitudinal sections, were examined to help identify fracture types. Figure 1 illustrates the alloy production, hydrogen-charging, mechanical testing, and compositional checking.

**Figure 1:**
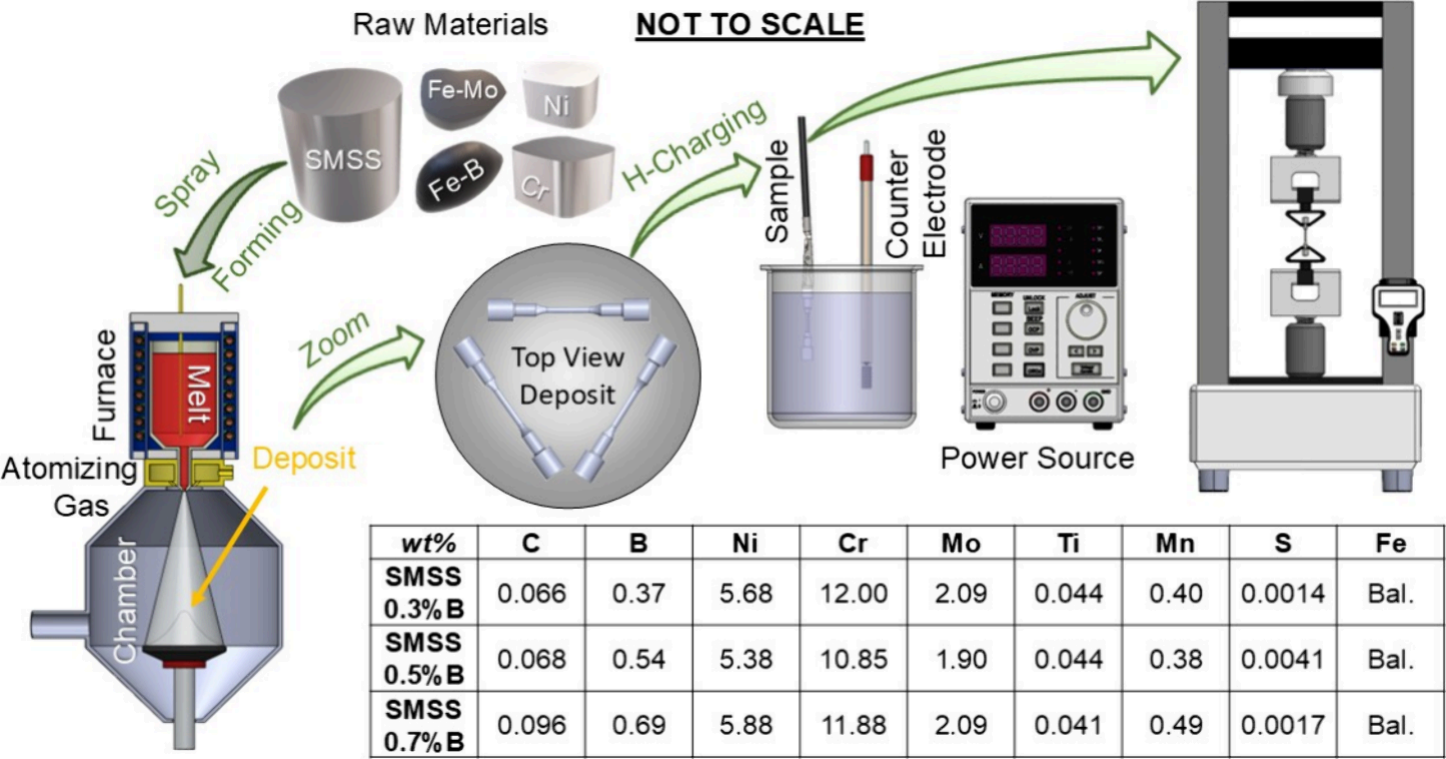
Illustration of the production process for boron-modified super martensitic stainless steel (SMSS) using spray forming, followed by hydrogen charging and tensile testing. The inset table presents the elemental composition of the as-deposited SMSS with varying boron contents.

## 3 Results and Discussion

Figure 2a–d illustrates the microstructures of the commercial and spray-formed SMSS with 0.3%B, 0.5%B, and 0.7%B alloys. The addition of boron leads to the formation of equiaxed martensitic grains surrounded by a network of eutectic M_2_B. The spray-forming process produced refined equiaxed martensitic grains, with grain size decreasing progressively with increasing boron content (Figure 2b–d). While grain refinement typically enhances strength, the increased grain boundary area may also provide additional pathways for hydrogen diffusion and accumulation, potentially exacerbating the HE susceptibility. The alloy with the highest boron content (SMSS+0.7B) exhibits thicker borides and finer grains, indicating that boron promotes both grain refinement and boride network thickening. While minor variations in carbon and chromium content exist between the alloys (as necessitated by compositional balancing during boron addition), these differences (Figure 1) are insufficient to significantly alter the mechanical properties compared to the dominant effects of boron content and boride morphology. Figure 2e presents the XRD patterns, revealing the martensitic phase as the primary matrix, with boride peaks exhibiting low intensity, complicating their identification. Importantly, no hydride formation was detected in the hydrogenated samples, which suggests that either the alloying and processing conditions suppressed hydride precipitation or the quantity was below the detection limit of XRD.

**Figure 2:**
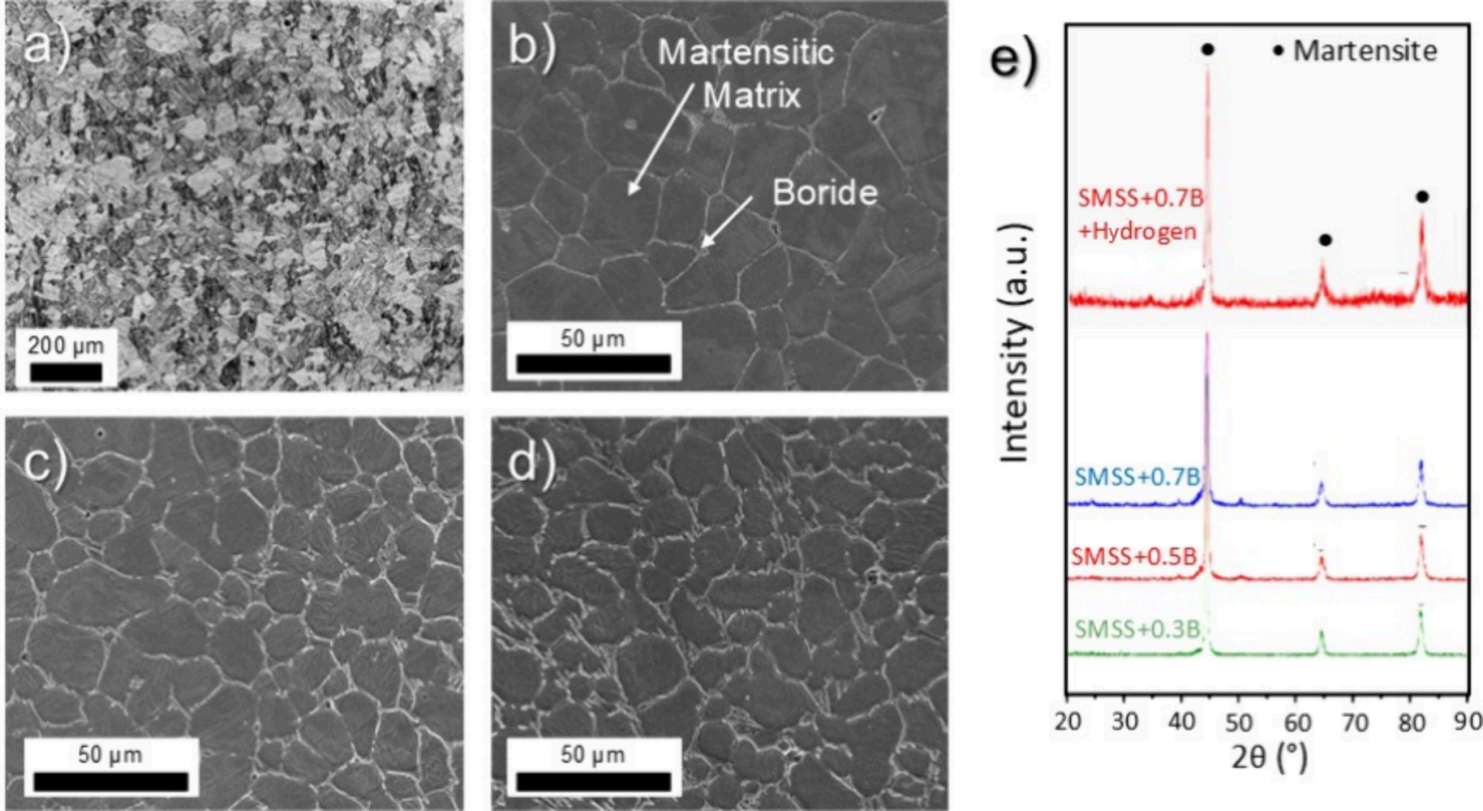
SEM microstructure of (a) SMSS, (b) SMSS+0.3%B, (c) SMSS+0.5%B, and (d) SMSS+0.7%B spray-formed alloys. Etching: Aqua regia. (e) XRD patterns, with the result of SMSS+0.7B after hydrogenation included for comparison.

Figure 3a depicts the tensile curves comparing hydrogenated and nonhydrogenated conditions. All boron-modified SMSS alloys exhibited higher ultimate tensile strength (0.3B–1200 MPa, 0.5B–1350 MPa, 0.7B–1530 MPa) compared to hot-rolled SMSS (850 MPa), albeit with reduced elongation to fracture. The enhanced tensile strength is attributed to the robust boride network at grain boundaries, which acts as a barrier to the dislocation motion. Additionally, the spray-forming process contributes to the formation of a fine, equiaxed microstructure, which is known to enhance the strength. However, the brittle nature of borides and their percolated structure severely compromises ductility, with elongation to fracture ranging from 3 to 3.7%, significantly lower than the ∼12% observed in commercial hot-rolled SMSS. The latter demonstrates significant necking (Figure 3b) and dimple formation (Figure 3c), indicative of ductile fracture. In contrast, fracture analysis of boron-containing SMSS reveals minimal necking (Figure 3d,g,j) and brittle fracture behavior with negligible plastic deformation (Figure 3e,h,l). Crack propagation predominantly occurs by cleavage of the martensitic matrix and along boride-decorated grain boundaries (Figure 3f,i,k), highlighting the detrimental role of borides in fracture toughness.

**Figure 3:**
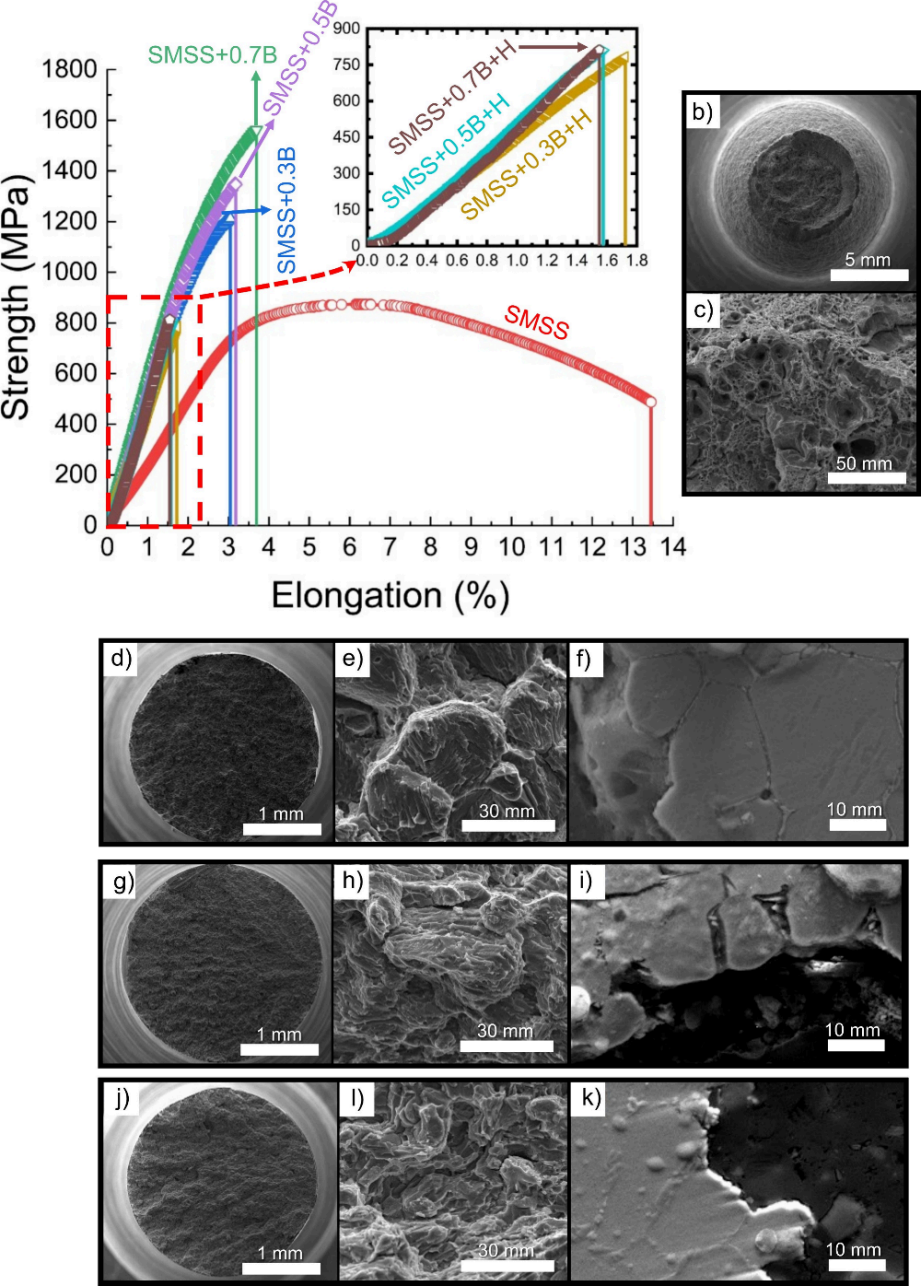
(a) Tensile curves of spray-formed alloys with varying boron contents tested with and without hydrogenation, including SMSS as a reference. The inset highlights the behavior of hydrogenated samples. (b, c) SEM-SE images of the fractured surface of SMSS without hydrogenation. SEM-SE images of the fractured surface of SMSS, without hydrogenation, containing different addition of boron of: (d, e) SEM-SE images of SMSS+0.3%B, with (f) showing the polished and etched cross-section, (g, h) SEM-SE images of SMSS+0.5%B, with (i) showing the polished and etched cross-section. (j, l) SEM-SE images of SMSS+0.7%B, with (k) showing the polished and etched cross-section. Etchant: aqua regia.

Hydrogen charging resulted in a pronounced reduction in tensile strength (33–41%) and elongation to fracture, regardless of boron content. All hydrogenated materials exhibited similar failure stress levels (∼800 MPa) and elongation to fractures around 1.6%, irrespective of their boron content. Post-tensile test hydrogen measurements indicated hydrogen concentrations, in weight, of 10, 17, and 18 ppm for SMSS+0.3%B, SMSS+0.5%B, and SMSS+0.7%B, respectively. These findings underscore the susceptibility of boron-containing SMSS to HE, as well as, if any, a limited influence of boron content on mitigating hydrogen’s detrimental effects on the matrix.

While this study focused on boron-modified SMSS, comparative analysis with conventional alloying approaches reveals critical trade-offs: the baseline 13Cr-5Ni-2Mo SMSS showed a 44.5% elongation reduction after hydrogen charging,^13^ whereas boron-modified alloys achieved higher strength (∼1530 MPa for SMSS+0.7%B) but suffered extreme HE susceptibility (1.6% elongation) due to M_2_B-induced intergranular cracking. This contrasts sharply with Ni/Mo-stabilized systems, where precipitated austenite reduces hydrogen diffusion,^13^ and Mn-modified grades, where TRIP effects improve HE resistance by 40%.^26^ While Nb additions could theoretically enhance HE resistance through carbide trapping,^27,28^ our results demonstrate that boron’s strength benefits are overwhelmingly offset by its promotion of brittle fracture pathways—a more severe compromise than observed in conventional SMSS variants. These findings highlight an unresolved materials design challenge: boron’s exceptional strengthening effect is inherently coupled with acute HE vulnerability, suggesting that future work should explore hybrid approaches combining controlled boron additions with austenite-stabilizing elements to decouple strength from embrittlement. Compared to boron-free SMSS, the boride-reinforced SMSS are more prone to HE.

The pronounced hydrogen embrittlement (HE) susceptibility observed in boron-modified SMSS raises an important question: can coalloying with elements such as Ti or Nb mitigate embrittlement while preserving the strength gains imparted by boron? Evidence from related systems supports this possibility. In hot-stamped boron steels, minor Ti additions (∼0.03 wt %) markedly improved HE resistance by promoting the formation of nanoscale TiC precipitates that serve as irreversible hydrogen traps, effectively reducing hydrogen mobility while maintaining high strength (∼1500 MPa).^29^ Similarly, Nb additions have been shown to inhibit hydrogen blistering in pipeline steels by facilitating the formation of fine NbC precipitates, which homogenize hydrogen distribution and suppress crack nucleation.^30^ Notably, synergistic Nb–Ti coalloying strategies in hot-stamped boron steels achieved even greater improvements: nanosized (Nb,Ti)C precipitates became the dominant hydrogen trap sites over grain boundaries, reducing hydrogen diffusivity by approximately 40% and proportionally decreasing HE susceptibility with increasing Nb+Ti content.^31^ Extrapolating from these findings, strategic coalloying in boron-modified SMSS could potentially disrupt the continuous M_2_B boride networks through: (1) introducing fine (Ti,Nb)C precipitates as alternative nucleation sites during solidification, (2) providing strong hydrogen traps to mitigate grain boundary hydrogen accumulation, and (3) refining boride morphology to decrease intergranular cracking pathways. Although such coalloying approaches were beyond the scope of the present study, future work should systematically explore the effects of incorporating 0.03–0.1 wt % Ti and/or Nb alongside 0.2–0.5 wt % B to evaluate whether tailored precipitate engineering can successfully decouple the strength/embrittlement trade-off—a crucial step toward realizing high-strength SMSS capable of operating in hydrogen-containing environments.

Figure 4a–i presents the fractured surfaces of hydrogenated alloys, revealing pronounced embrittlement compared to their nonhydrogenated counterparts (Figure 3d–k). The fractures are predominantly intergranular with evident branching, and borides decorating the grain boundaries frequently exhibit cleavage. Figure 4c,f,i shows longitudinal sections of the samples, providing additional insights into the fracture mechanisms. Similar to nonhydrogenated samples, the fractures remain predominantly intergranular; however, hydrogenated samples display secondary cracks and intergranular fractures extending below the fracture surface and along the gauge length of the specimen. This observation indicates a more pervasive embrittlement effect driven by hydrogen diffusion and accumulation at material interfaces.

**Figure 4:**
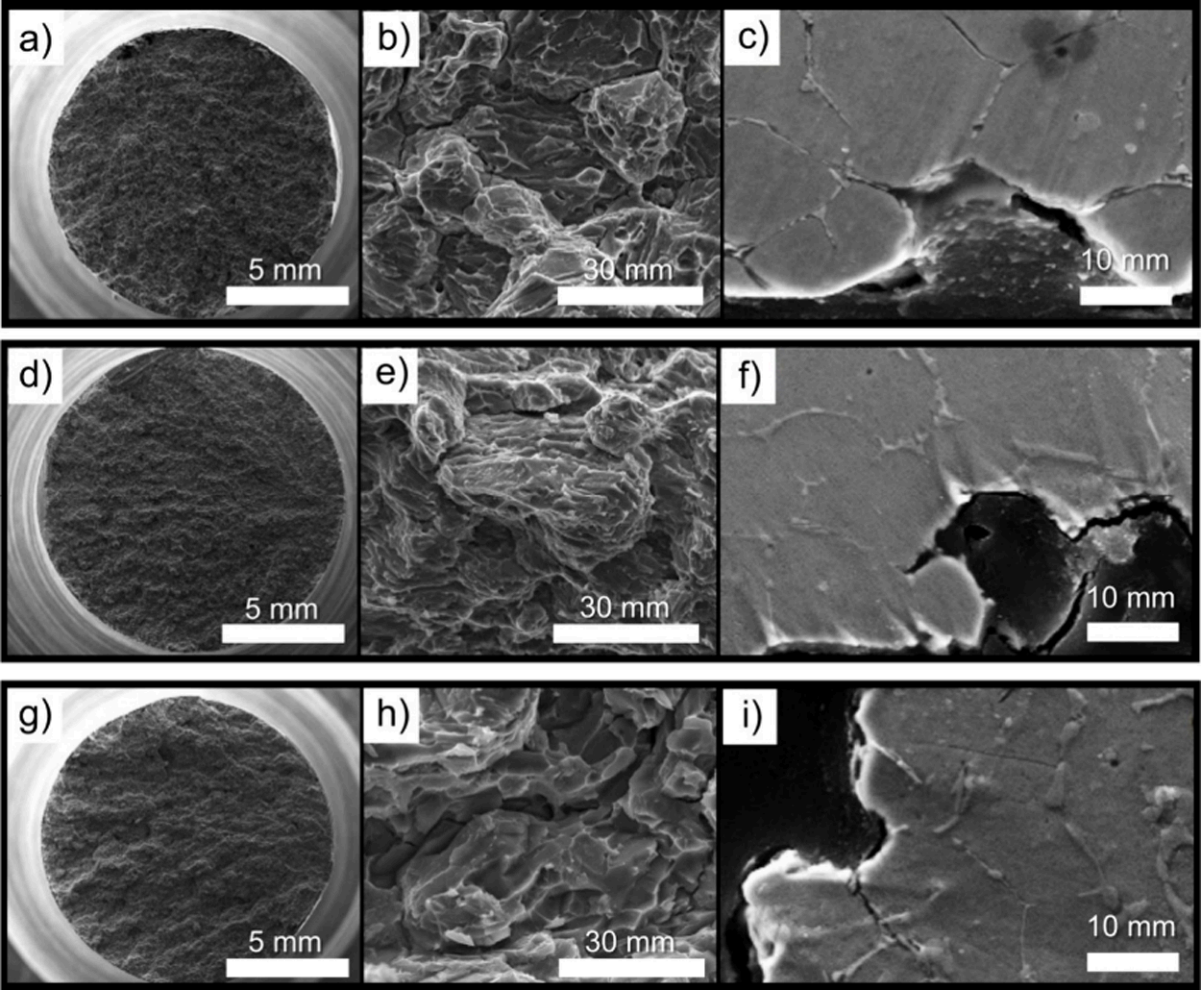
SEM-SE images of the fractured surface of SMSS with hydrogenation containing different addition of boron of (a, b) SMSS+0.3%B, with (c) showing a polished and etched cross-sectional view; (d, e) SMSS+0.5%B, with (f) showing a polished and etched cross-sectional view; and (g, h) SEM-SE images of SMSS+0.7%B, with (i) showing a polished and etched cross-sectional view. Etchant: aqua regia.

The morphology (size, distribution, and continuity) of borides plays a central role in HE. The percolated M_2_B network, which becomes more continuous and thicker with increasing boron content (Figure 2), acts as both a strengthening phase and a preferential pathway for hydrogen-assisted cracking. The brittle borides reinforce grain boundaries, enhancing strength; however, they also serve as hydrogen trapping sites and crack initiation points due to stress concentrations at matrix–boride interfaces. Furthermore, the interconnected boride distribution dictates crack propagation—more continuous networks facilitate intergranular fracture by linking microcracks along grain boundaries, exacerbating HE susceptibility.

SMSS, even without boron addition, is inherently sensitive to HE, as evidenced by a reported 44.5% reduction in elongation to fracture for hydrogenated samples compared to nonhydrogenated ones.^13^ The physical and chemical characteristics of material interfaces—including grain boundaries, twin boundaries, and matrix/precipitate interfaces—play a crucial role in determining susceptibility to HE.^32^ In the context of boron-containing SMSS, boride-decorated grain boundaries exhibit heightened vulnerability to intergranular fractures under hydrogen exposure. This heightened sensitivity can be attributed to the interaction of hydrogen with the borides and the stress concentration at these interfaces, further exacerbating the embrittlement.

Overall, the findings emphasize a trade-off between strength and ductility in boron-modified SMSS and highlight the critical role of hydrogen in exacerbating intergranular fracture. The apparent independence of elongation-to-fracture from total hydrogen content (despite variations in boride morphology) suggests that HE is governed by local hydrogen saturation at critical microstructural features rather than by bulk concentration. Future work should explore threshold hydrogen concentrations and microstructural design strategies to disrupt percolated boride networks, thereby improving HE resistance without sacrificing strength.

## 4 Conclusions

- The tensile strength of SMSS-0.5%B and SMSS-0.7%B alloys is similar and higher than that of SMSS-0.3%B due to the smaller grain sizes of the former and the larger boride content, which contribute to their superior mechanical properties.
- The addition of boron via the spray-forming process enhances the tensile strength of supermartensitic steels but leads to a significant reduction in ductility. This trend is further exacerbated by hydrogen exposure, which further lowers both tensile strength and elongation.
- HE exhibits a pronounced effect over time, with samples subjected to hydrogenation for more than 168 h showing over a 55% reduction in mechanical properties, particularly in tensile strength and elongation to fracture.
- Regardless of hydrogen exposure, spray-formed boron-containing supermartensitic stainless steels tend to exhibit brittle, intergranular fracture behavior.
- Both the martensitic matrices and borides are susceptible to HE. The brittleness of M_2_B-type borides, which form along the grain boundaries, facilitates intergranular fracture. Following embrittlement of these grain boundaries, secondary cracks may propagate through the martensitic matrix, supporting the hypothesis that HE involves both the boride and the martensitic matrix.
